# Men Who Compliment a Woman's Appearance Using Metaphorical Language: Associations with Creativity, Masculinity, Intelligence and Attractiveness

**DOI:** 10.3389/fpsyg.2017.02185

**Published:** 2017-12-21

**Authors:** Zhao Gao, Qi Yang, Xiaole Ma, Benjamin Becker, Keshuang Li, Feng Zhou, Keith M. Kendrick

**Affiliations:** ^1^School of Life Science and Technology, University of Electronic Science and Technology of China, Chengdu, China; ^2^School of Foreign Languages, University of Electronic Science and Technology of China, Chengdu, China; ^3^Key Laboratory for Neuroinformation, Center for Information in Medicine, University of Electronic Science and Technology of China, Chengdu, China

**Keywords:** metaphor, creativity, mate selection, 2D:4D ratio, masculinity

## Abstract

Language may have evolved as a signal of mental fitness. However, it remains unclear what language form and topic men use to covertly signal mate quality. In this study 69 men created compliments to impress unfamiliar women they chose to either date or work with and provided hand scans to compute 2D4D ratio as a proxy for prenatal testosterone exposure and masculinity indicator. Compliments were coded in terms of form (literal vs. metaphorical) and topic (women's appearance vs. non-appearance), with metaphorical ones being subsequently rated by 114 women for psycholinguistic features, indices of intelligence and willingness to have a romantic relationship with the author. Results showed that in a dating context, men produced more metaphorical form compliments targeting appearance compared to the working context and they were associated with men's art creativity and negatively with 2D4D ratio (i.e., positively with masculinity). Women preferred establishing a romantic relationship with a higher proportion of the men producing metaphorical compliments in a dating than a working context. Furthermore, in the dating but not the working context, women perceived men producing such compliments as being more intelligent, and importantly this correlated with the men's actual verbal intelligence. Overall, findings suggest that men may use metaphorical language compliments targeting women's appearance in a dating context to signal covertly their mate quality.

## Introduction

Scheherazade in *The Arabian Nights* finally won the heart of the king solely by telling him stories for 1,001 nights, which displayed both her creativity and humor. Miller (2000) used this analogy as the basis for his “Scheherazade hypothesis” which posits that the evolution of complex language may have been influenced by the need to be more attractive to potential mates and to maintain their subsequent interest. Language fulfills a number of both social and non-social functions which may all have contributed in some way to its evolution (Dunbar, 2003), but if specific aspects have evolved to signal mate quality then they should be both reliable and difficult to counterfeit (Rosenberg and Tunney, 2008; Lange, 2011; Hall, 2015). The use of language in a courtship context can be both complex and subtle (Gersick and Kurzban, 2014), and therefore the specific form and content of the “good gene” signals in courtship language might also be subtle and covert in nature (Grice, 1975; Miller, 2000; Gersick and Kurzban, 2014).

While flirtation utilizes a combination of both non-verbal and verbal communication and involve different styles (Hall and Xing, 2015) one of the most important verbal communication strategies is paying compliments. Paying compliments to others represents a ubiquitous flirting strategy to both start and maintain a romantic relationship. Compliments can transmit sexual interest in a socially acceptable form of speech, without taking any “socially imposed risks” such as face threat (Holmes, 1986; Doohan and Manusov, 2004; Rees-Miller, 2011; Gersick and Kurzban, 2014), while simultaneously increasing the probability of a positive affective response from the receiver (Bale et al., 2006; Cooper et al., 2007; Brown et al., 2014). The topic of compliments can be sex- or context- dependent and able to indicate sexual interest during interpersonal interactions (Doohan and Manusov, 2004; Rees-Miller, 2011; Brown et al., 2014). For example, appearance is the most frequent compliment topic in a romantic context, followed by personality (Doohan and Manusov, 2004). It is well established that across cultures men focus more on visual physical attributes when judging attractiveness in women (Buss, 1989), and indeed a social impression can be generated after a face is seen for only around a 100 ms (Todorov et al., 2009). There is also a positive association between the secondary sexual characteristics in faces and immunocompetence which has influenced the perceived attractiveness of feminine faces in women and masculine ones in men and the content of chat-up lines in form of compliments, particularly when a short-term relationship is pursued (Little et al., 2011). Thus, men's compliments on women's appearance can be considered as being flirtatious, indicating sexual interest (Parisi and Wogan, 2006). However, few studies have specifically investigated whether the compliment topic employed by men and its sexual intent can signal mate quality.

In addition to the choice of topic in a compliment, its expressive complexity may also reveal mate qualities such as the cognitive or social intelligence of the individual producing it (Miller, 2000; Ball et al., 2007; Rosenberg and Tunney, 2008; Sampson et al., 2009; Gersick and Kurzban, 2014; Lange and Euler, 2014). Metaphor, for example, is regarded as a more complex language form than literalness (Cacciari, 1998; Glucksberg, 1998) since it involves higher-order cognitive computation (Eviatar and Just, 2006), cross-domain projection of concepts (Fauconnier and Turner, 1998) and more extensive involvement of both language and cognitive brain networks (Benedek et al., 2014). The semantic complexity that is achieved through conceptual blending generates both novel meaning and increased aesthetic appreciation (Rolls, 2017), and even enhances the attractiveness of metaphorical language users (Gao et al., 2017). The creative ability exhibited in metaphorical compliments might signal reproductive fitness in terms of superior intellectual skills and therefore contribute toward the costly evolution of higher level linguistic abilities (Rosenberg and Tunney, 2008; Lange, 2011; Lange and Euler, 2014; Donahue and Green, 2016). However, it remains unclear whether or not male language users intentionally display their prospective mate qualities via metaphor production when paying compliments to women. If they do indeed use this strategy to display explicitly their mate quality, then they should do so more in a mating context than in other social contexts where they do not intend to convey any romantic or sexual interest.

Given the well-established influence of gonadal hormones on human social, cognitive and emotional behaviors (Bos et al., 2010; Coyle and Kaschak, 2012; Decety and Svetlova, 2012), androgens might also influence male language development (Beech and Beauvois, 2006; Albores-Gallo et al., 2009; Kung et al., 2016). Prenatal testosterone (pT) for example influences the sexual differentiation of the brain, including lateralization of language representation, and high levels may even contribute to the development of autistic traits and associated language impairments (Baron-Cohen, 2002; Lutchmaya et al., 2004; Auyeung et al., 2009; Lust et al., 2010; Bosch-Domènech et al., 2014; Hahn et al., 2016). However, the relationship between pT and language ability remains controversial (Papadatou-Pastou and Martin, 2017). Higher pT concentrations are also associated with more masculine behavior (Aromäki et al., 2002; Bancroft, 2005; Auyeung et al., 2009; Welker et al., 2014; Kung et al., 2016; Mailhos et al., 2016; Atkinson et al., 2017), indicative of a man's physical and reproductive health (Johnston et al., 2001; Klimek et al., 2014), and a stronger sex drive (Aromäki et al., 2002). Thus, the propensity for men to use language to display their mate quality might vary as a function of individual differences in pT levels. Due to the influence of pT on fetal cartilage development it can influence the ratio of second-to-fourth digit length (2D:4D ratio) and this is commonly measured as an indirect somatic marker for pT, particularly the right-hand ratio (Manning et al., 2014). The higher the pT level an individual is exposed to, and lower estrogen, in the fetal environment, the lower 2D4D ratio they have (Lutchmaya et al., 2004), and the more masculine type behavior they display. Furthermore, women rate men with low 2D4D ratio's as more attractive after short direct interaction encounters where obviously language communication as well as other physical features are involved (Roney and Maestripieri, 2004).

Against this background, the present study aimed to address the question of how men may both display their mate qualities and communicate sexual interest through the form and content of the verbal compliments that they pay to women in a mating vs. non-mating context. To this end, two different contexts—dating vs. working—were created, the former of which encourages the explicit behavior of mate selection whereas the latter does not. Male participants were required to produce compliments to impress the women they chose to either work or date with. Based on prior literature discussed above, we firstly hypothesized that in a dating context, which has a strong sexual component relative to a working one, men would tend to produce more and better quality metaphorical expression compliments toward women than literal ones and which primarily targeted their appearance, reflecting their sexual intention. In a secondary more exploratory analysis which focused specifically on the characteristics of men who produced the compliments we hypothesized that those displaying the highest frequency of metaphorical compliments targeting a woman's appearance in a dating context would also be the most masculine (in terms of their 2D4D ratio) and creative. Finally, we hypothesized that if women could interpret mate-quality signals from the compliments produced in a dating context, they would be more likely to choose to have a relationship with men producing metaphorical compliments targeting their appearance in the dating than the working context and perceive them as being more intelligent.

## Materials and methods

### Participants

Sixty-nine male university students (mean age = 20.86, SE = 0.272) were recruited. In order to guarantee the homogeneity of two groups of participants, four kinds of questionnaires and tests were administered before the experiment: (1) basic psychological state: Social Esteem Scale (SES; Cronbach's α = 0.857) (Rosenberg, 1965), Cheek and Buss Shyness Scale (CBSS; Cronbach's α = 0.813) (Cheek, Unpublished Manuscript), Beck Depression Inventory (BDI; Cronbach's α = 0.838) (Beck et al., 1996); (2) creative ability: Kaufman Domains of Creativity Scale-Chinese Revised (K-DOCS; Cronbach's α = 0.903 with lowest subscale 0.698) (Tu and Fan, 2015), (3) IQ: general IQ assessed using Raven's Standard Progressive Matrices—Chinese Revised (R SPM) (Zhang and Wang, 1989) and verbal IQ assessed using the vocabulary test of Weschler's Intelligence Scale-Chinese Revised (WAIS-R) (Gong, 1983); (4) love experience: Love Attitude Scale (LAS) (Hendrick et al., 1999) [subscale Cronbach's α values between 0.706 and 0.818; (Wu and Wu, 2005; Yang et al., 2008)], and Passionate Love Scale (PLS) (Hatfield and Sprecher, 1986); (5) Chinese language proficiency: score in the National College Entrance Exam (CEE-Chinese). The *CEE-Chinese* has a score of up to 150 in the National College Entrance Exam and is the standard exam for Chinese language proficiency used nationwide. A demographic questionnaire included age, years of education, sexual orientation, current relationship status, number of relationships and their duration, and the motivation to find a new romantic partner. In line with the specific hypotheses in our study only group differences in relation to creativity and IQ were analyzed in the context of the compliments produced by male subjects. Although approximately half of the subjects currently had a girlfriend their motivation to find a new one and passion for love (in terms of PLS score) was not significantly different from subjects who currently did not have one, and so data from them were combined (see Table 1 and Tables S1–S3). Indeed, in general subjects expressed only a moderate desire to find a girlfriend, which may reflect their current student status and the importance of other factors such as their studies. Six participants were excluded due to either having clinically relevant depression levels (*N* = 1, BDI score = 29) or misunderstanding task instructions (*N* = 5). Additionally, 114 female university students (*M*_age_ = 20.74, SE = 0.171, *N* = 114) were recruited to rate the psychological, linguistic and other qualities of the compliments produced by the male subjects, and for those who were currently single (*n* = 68) to rate whether they would like to have either a short or long-term relationship with the author.

**Table 1:** Groups of male participants in the two contexts.

**All participants**	**Dating Context (*N* = 31)**		**Working Context (*N* = 32)**		***t***	***p***
	**Mean**	**SE**	**Mean**	**SE**		
**DEMOGRAPHIC INFORMATION**						
Age (years)	21.39	0.39	20.47	0.39	1.67	0.10
2D4D Ratio (right hand)	0.95	0.01	0.96	<0.01	−0.23	0.82
2D4D Ratio (left hand)	0.95	0.01	0.96	0.01	−0.66	0.51
Education (years)	15.06	0.34	15.14	0.33	0.23	0.82
R SPM	50.78	1.35	49.03	1.63	0.82	0.42
CBSS	33.00	1.29	36.88	1.72	−1.79	0.08
BDI	7.40	0.91	9.44	1.21	−1.32	0.19
SES	31.19	0.85	30.28	0.74	0.81	0.42
Intensity of love (PLS score)	107.97	2.59	104.47	2.25	1.02	0.31
Longest relationship (months)	17.45	3.70	10.75	3.19	1.37	0.17
Love times^a^	Mean rank: 36.08		Mean rank: 28.05		Z = −0.83	0.07
**KDOCS CREATIVITY**						
KDOCS art	27.94	1.14	27.53	0.85	0.29	0.78
KDOCS everyday	38.48	1.19	36.47	0.80	1.42	0.16
KDOCS performance	27.94	1.37	24.19	1.50	1.84	0.07
KDOCS scholarly	32.32	1.12	32.94	1.02	0.26	0.80
KDOCS science	31.58	1.31	30.06	1.04	0.91	0.37
**CHINESE LANGUAGE TEST ^b^**						
CEE-Chinese	113.14	1.58	112.94	1.34	0.12	0.90
Chinese Writing Test (CWT)	10.40	0.25	10.38	0.25	0.08	0.94
Romance of Encounter Description	4.70	0.15	3.13	0.22	**5.92**^c^	^***^
WAIS Vocabulary Subscale	11.74	0.25	11.29	0.22	1.34	0.19

a *Mann–Whitney test results*.

b *CEE–Chinese was for Chinese Entrance Exam-Chinese, Chinese score in the National College Entrance Exam, which was up to 150. Two writing tasks were administered sequentially prior to experiments: Chinese Writing Test (CWT) in which a short story was to be continued for 15 minutes and account on imaginary encounter for 12 minutes. Romance of Encounter Description was measured on a 7-point scale. WAIS Vocabulary Subscale was the vocabulary test of WAIS VIQ*.

c *The language descriptions on romantic encounter in the dating context were rated significantly higher than that in the working context*.

*** *Significance level at p < 0.001 two-tailed*.

The present study had full ethical approval from the local ethics committee at the University of Electronic Science and Technology of China and was in accordance with the latest revision of the Declaration of Helsinki. Every participant signed informed consent forms before the experiment, and was paid 70 CNY at the end of experiment.

### Experimental procedure

The experimental sessions lasted a total of approximately 3 h. Firstly, baseline language proficiency for each of the 63 participants was established by administering the vocabulary test of the Wechsler Intelligence Scale and a 15-min Chinese writing test. The Chinese writing test in this study was part of a paper sample of HANYU NENGLI CESHI Band Five (HNC-5), which is a national Chinese proficiency test for college-level native speakers. In the writing task, subjects were required to continue on from the beginning of a short story. Subjects also completed the other questionnaires included in the study and both of their hand palms scanned and the images stored as JPEG pictures in order to analyze 2D4D ratios. Participants were then presented with high-resolution color photographs of 30 unfamiliar, young and attractive women which were trimmed to only include the shoulders, neck and head. Photographs were standardized as 433 × 509 pixels (646 KB) and rated them in terms of agreeableness (*M* = 4.88, SE = 0.46), attractiveness (*M* = 5.55, SE = 0.04), arousal (*M* = 5.55, SE = 0.04), valence (*M* = 5.08, SE = 0.06), and intelligence (*M* = 5.20, SE = 0.03) on a 7-point scale. Next, participants were randomly assigned to either produce compliments in a “dating” (*n* = 31) or “working” (*n* = 32) context (see File S1 for the different context descriptions provided) and asked to choose the photographs of 4 out of the 30 women who they most wanted to interact with in that specific context. This procedure both allowed for individual differences in selection and, importantly also enhanced the credibility of the paradigm. The distribution of choices of the 30 different pictures and the average ratings for them are provided in Table S4. Out of the 30 pictures, only 4 were chosen at a significantly different frequency in the two contexts. Three of these chosen disproportionately in the dating context were rated as more attractive but a further analysis revealed that they did not contribute significantly to group differences in compliment production/topic (see Table S5).

After a short break, the main part of the experiment involved the participants imagining an encounter with one or all of the 4 women they had chosen and writing down the details of it in order to establish that the romantic context had been successfully primed (12 min). Participants were then asked to complete two written tasks to promote their compliment production: a free writing and a completion task (Taylor, 1947; Pierce and Chiappe, 2008). For the 10-min free writing task, participants wrote down complimentary sentences in order to impress the women they selected. For the completion task, which had no time limit, participants filled in blanks in a standard structure for compliments: “Your ___(*n*.) is/are ___(*adj*.) ___(*n*.)” (i.e., “Your eyes are shining stars”).

### Coding and rating of compliments

A coding scheme was created as a guideline for sentence categorization (Rees-Miller, 2011). The appearance topic compliments were divided into those targeting face, hair, lip, smile, eyes, voice and other parts of the body, etc., whereas the non-appearance topic category was divided into personality, temperament, mind or other general descriptions that did not involve physical appearance (see Table S6). No compliments were produced which targeted overtly sexual features such as breasts and buttocks and which might be construed as highly inappropriate in a working context. In each case the compliments produced were also coded as either literal or metaphorical. Two research assistants studying Chinese language independently coded all the sentences based on this coding scheme (inter-rater correlation of 0.804~0.895 (Spearman's rho), *p*s < 0.001). In line with the major research question of the present study, compliments produced were combined into four general categories: metaphorical expressions targeting appearance (M-A), metaphorical expressions targeting non-appearance (M-NA), literal expressions targeting appearance (L-A) and literal expressions targeting non-appearance (L-NA). A total of 560 metaphorical compliments produced in the completion task (excluding identical ones) were prepared for the 114 women raters.

Given the large number of compliments and different scales which required rating we decided to divide the 114 women raters into 4 different groups each receiving a random selection of different metaphorical compliments to rate. The women in all four groups also rated a random sub-group of the same 18 metaphorical compliments in order to confirm inter-group consistency (inter-group rating consistency was high—see Table S7 and so raters were considered as a homogeneous single group). The features of metaphorical language were rated on 7-point scales in terms of basic relevance (appropriateness, valence), language dimension (figurativeness, familiarity, imageability) (Cardillo et al., 2010), emotional dimension (arousal, romance, attractiveness), and interpersonal impression (perceived intelligence). Thus metaphorical language compliments created by male participants were rated as being appropriate in a real life situation and having a positive valence from 1(= not at all) to 7 (= very much). Next, the linguistic quality of each compliment produced was rated in terms of figurativeness from 1 (very literal) to 7 (very figurative), imageability measuring how quickly and easily a sentence activates a visual mental image from 1 (= very slowly and with difficulty) to 7 (= very quickly and easily), and familiarity measuring the frequency of an expression from 1 (= very unusual) to 7 (= very familiar). In addition, the emotional response evoked by each compliment was rated in terms of arousal, romance and attraction from 1 (= not at all) to 7 (very much). Finally, the potential for the compliments produced to act successfully as “mate quality” signals was further explored. For each metaphorical compliment the single women raters were asked to indicate what kind of relationship they would like to have with the male writer (romantic partner for either a short- or long-term relationship; a friend; none) and how intelligent they perceived them to be (1 = not intelligent to 7 very intelligent) based entirely on the language used. The tasks were all presented using Eprime 2.0 on a computer screen.

The Chinese writing tests given to the male participants which were used to test Chinese language proficiency were graded between 0 and 20 points by two postgraduates majoring in linguistics and in accordance with the grading criteria of topic relevance, content, language expression, and punctuation marks in the HNC-5 Test Syllabus (inter-rater coefficient: Spearman rho = 0.805, *p* < 0.001). The accounts of the imaginary encounters provided by participants were also rated using a 7-point scale based on two female graders' subjective perception of work vs. romance (inter-rater coefficient: Spearman rho = 0.65, *p* < 0.001).

### Digit ratio measurement

The lengths of the second (2D) and fourth (4D) finger of both hands were measured by two independent research assistants using CorelDraw Graphics Suite X8 from hand palm photos scanned by an Epson V330 Photo scanner. The distance between the mid-point of the ventral proximal crease to the point of fingertip was used to determine the length of a digit. A high inter-rater reliability (Pearson *r*s: right hand = 0.87, left hand = 0.89, *p* < 0.001) and high intra-class correlations (right ICC = 0.93, *F* = 16.50, *p* < 0.001; left ICC = 0.92, *F* = 14.27, *p* < 0.001) for 2D:4D ratios were achieved.

### Data analysis

For a first analysis the number of compliments produced by each subject in the two contexts of the four different types (M-A; M-NA; L-A; L-NA) was used. A 2-way ANOVA analysis was then performed with group context as a between subject factor and compliment type as a within subject factor. *Post-hoc* tests using Bonferonni correction compared specific compliment types in the two contexts. Independent *t*-tests were used to compare women's ratings for the various qualities of the metaphorical compliments in the two contexts. For investigating associations between the different compliment types and male qualities (K-DOCS subscales and 2D4D ratio) the proportions of each type of compliment used were first calculated by expressing them as percentages of the total number produced by each subject. Pearson's correlation and a general linear regression model were then employed. Next, a Process analysis (Hayes, 2013) was used to explore the moderation effect of context and Fisher's *z*-test to explore between-context differences for every pair of correlations. Data from the two tasks for producing compliments (free writing and structured) were combined.

In order to analyze differences in choice of compliments by women in the two contexts the number of raters (single women only) who indicated that they wanted to have either a long or short term relationship with the male author or a friendship or no contact was first calculated for each compliment. Since our major interest was in terms of the choice of a romantic as opposed to a friendship interaction and very few compliments (0.05%) turned out to attract a selection of a long-term relationship with male writer, the long-term and short-term relationship options were combined into a single “romantic relationship” one. Any compliment where a greater proportion of raters chose to have a romantic relationship rather than friendship with the author was then considered as having successfully signaled a romantic interest. The proportion of male subjects who produced these successful compliments was then compared in the dating vs. working contexts using chi-squared.

The effect sizes for statistical results were calculated in each case: *r*^2^ for correlation analysis ($r_{\text{pb}}^{2}$ for point biserial correlation), $\eta_{p}^{2}$ for analysis of variance, Cohen's *d* for *t*-test, Cohen's *f* ^2^ for linear regression analysis, Cohen's *q* for Fisher's *z*-test, and Cohen's ω for Chi-square analysis.

## Results

No group differences were found between participants in the two contexts for language proficiency, or on any of the different categories of questionnaires given or for 2D4D ratios (see Table 1). Ratings for the description of the imagined encounter by participants were significantly higher for romance content in the dating context compared to the working one [*M*_dating_ ± SE = 4.70 ± 0.147, *M*_working_ ± SE = 3.13 ± 0.218, *t*_(60)_ = 5.996, *p* < 0.001, *d* = 1.508].

### Compliments produced by male participants in the dating and working contexts

**Figure 1:**
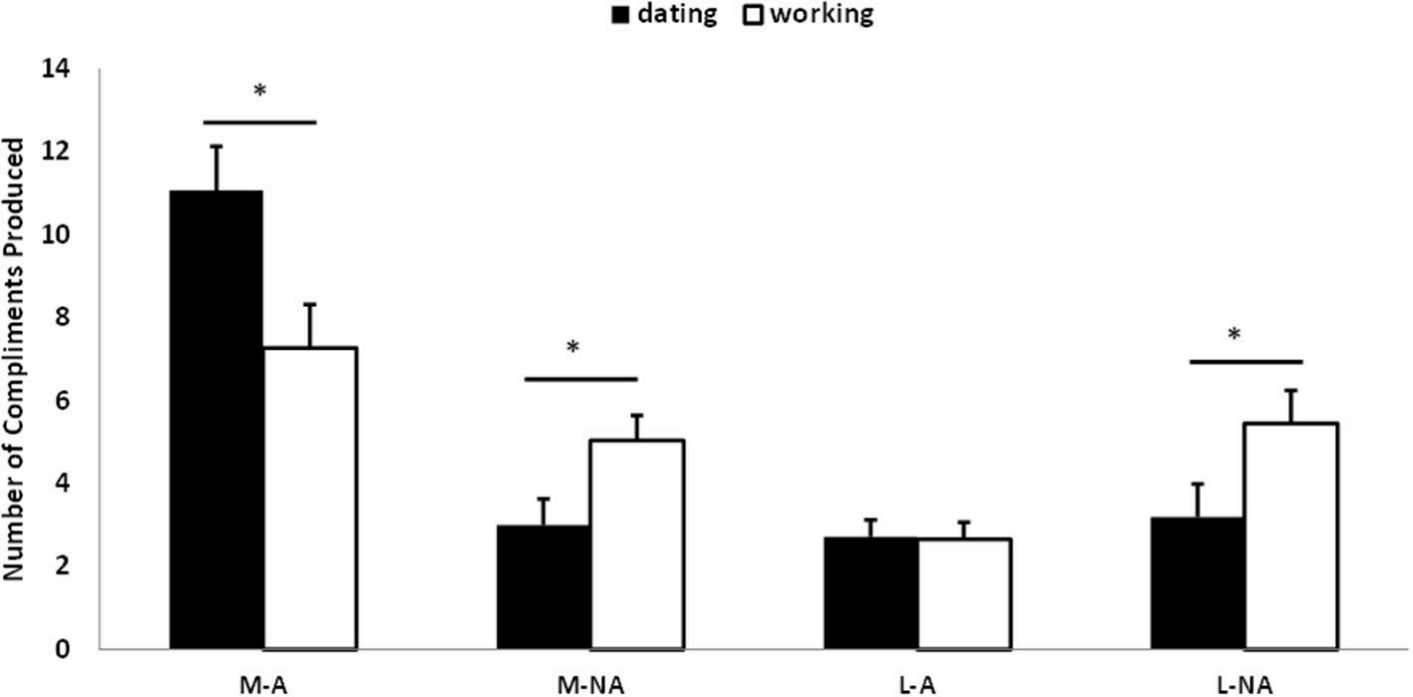
Interactive effect between context and compliment type. A greater number of metaphorical compliments targeting appearance were produced by male subjects in the dating context than in the working context whereas both literal and metaphorical compliments targeting nonappearance were produced more in the working context than in the dating context. No significant difference in literal expression targeting appearance was found between contexts. Bars show means and s.e., ^*^p < 0.05.

A mixed-design 2-way ANOVA with within-subject factor *compliment type* (M-A vs. M-NA vs. L-A vs. L-NA) and between-subject factor *contexts of language use* (dating vs. working) was conducted. The mean number of compliment sentences produced by the participants did not differ significantly in the two contexts [mean ± SE: dating context – 19.97 ± 1.39; working context – 20.41 ± 1.51, *t*_(61)_ = −0.213, *p* = 0.832, *d* = 0.055]. The ANOVA analysis revealed a significant main effect of compliment type [*F*_(3, 61)_ = 27.34, *p* < 0.001, $\eta_{p}^{2}$ = 0.309] and group context x compliment type interaction [*F*_(3, 61)_ = 6.688, *p* < 0.001, $\eta_{p}^{2}$ = 0.099; observed power = 97%]. Bonferroni corrected *post-hoc* analysis showed that the number of metaphorical compliments targeting appearance produced by men in the dating context was significantly greater than in the working context (mean ± SE – M-A_dating_ = 11.07 ± 1.07, M-A_working_ = 7.27 ± 1.05, *p* = 0.014, *d* = 0.639, observed power = 70.4%) whereas both metaphorical and literal compliments targeting non-appearance produced by men in the dating context were significantly smaller than in the working context (mean ± SE – M-NA_dating_ = 3.00 ± 0.62, M-NA_working_ = 5.03 ± 0.61, *p* = 0.023, *d* = 0.586, observed power = 62.8%; L-NA_dating_ = 3.19 ± 0.80, L-NA_working_ = 5.45 ± 0.79, *p* = 0.048, *d* = 0.508; observed power = 51%)(see Figure 1).

**Figure 2:**
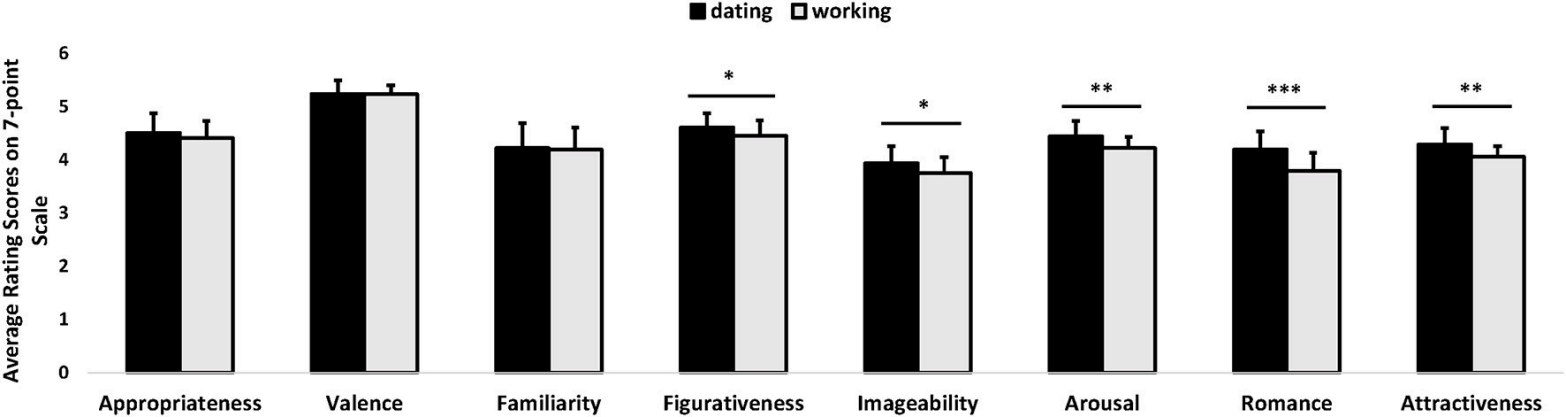
Women rated metaphorical compliments produced by male subjects in the dating context higher than in the working one across all criteria except for appropriateness, valence and familiarity. Bars show means and s.e., ^*^p < 0.05, ^**^p < 0.01, ^***^p < 0.001.

Independent *t*-tests showed that females rated the metaphorical compliments created by the male subjects in the dating context higher overall than those in the working context in terms of emotional responses (arousal, romance, attractiveness) and the key language features for metaphors (i.e., figurativeness, imageability) (all *p*s < 0.05). No significant differences were found for other features (appropriateness *p* = 0.301, *d* = 0.265; valence *p* = 0.862, *d* = 0.046; familiarity *p* = 0.811, *d* = 0.061) between the two contexts (see Figure 2). A breakdown of the different main appearance and non-appearance features complimented by the men in the two different contexts is given in Table S6 and importantly shows that there were no significant differences in the pattern of individual features targeted in the two contexts. Thus, it is unlikely that differences in the specific features men complimented women on in the two contexts could have contributed to observed differences in their perceived attractiveness,

### Associations between compliment language and men's characteristics, language ability and 2D4D ratios

The romance ratings for the imagined encounter descriptions composed by the male participants were positively correlated with the proportion of compliments targeting appearance (*r* = 0.394, *p* = 0.002, *r*^2^ = 0.155), those using a metaphorical form (*r* = 0.367, *p* = 0.003, *r*^2^ = 0.135) and also with higher figurativeness ratings (*r* = 0.304, *p* = 0.018, *r*^2^ = 0.092).

**Figure 3:**
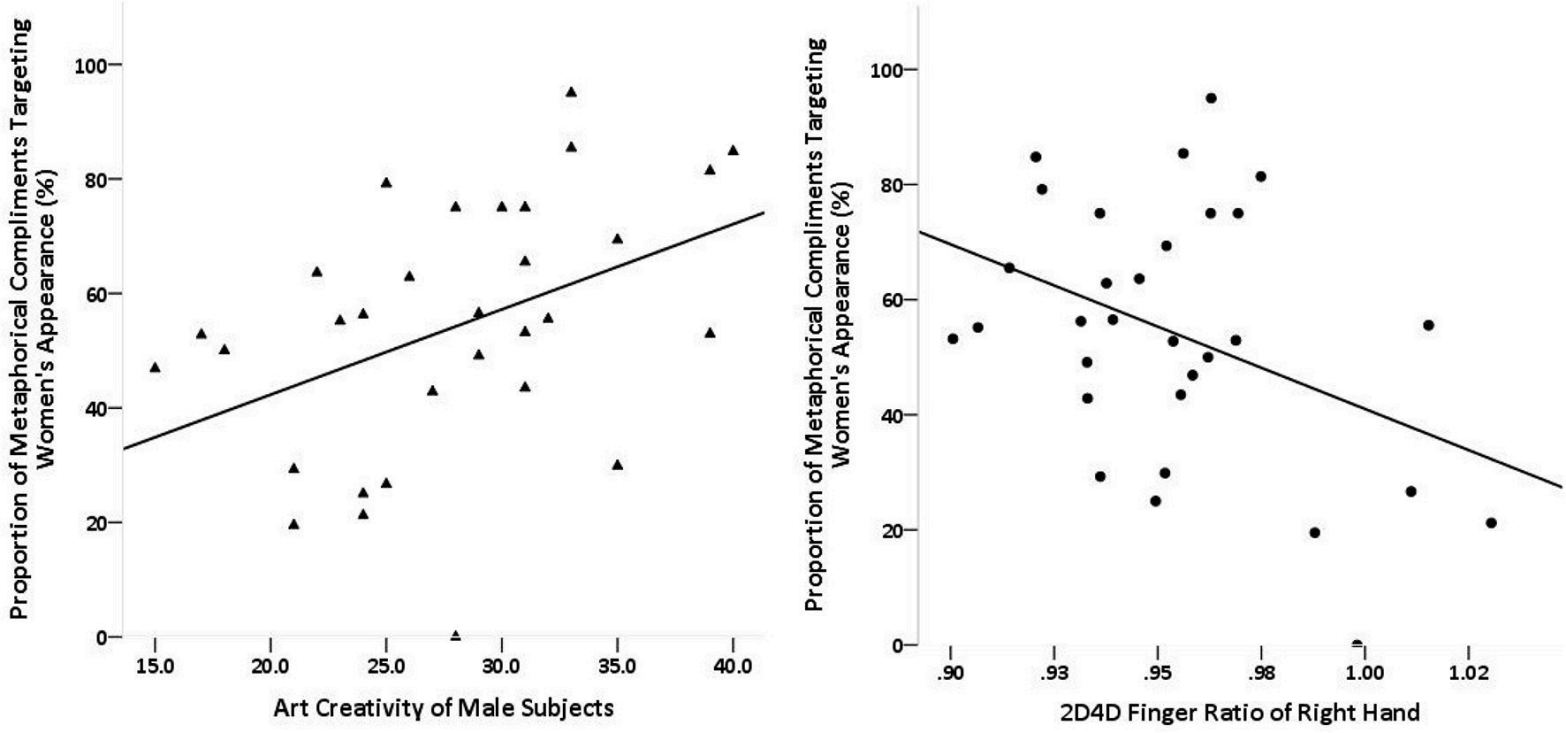
In the dating context the proportion of metaphorical compliments targeting women's appearance was positively correlated with the scores for K-DOCS art (**left**) *r* = 0.421, *p* = 0.018, and negatively correlated with the right-hand 2D4D ratio (**right**) *r* = −0.392, *p* = 0.029.

The association between the language of men's compliments and their mate qualities (2D4D ratio and creativity) was explored within each context. In the dating context the proportion of M-A compliments was positively correlated with the scores for K-DOCS art (*r* = 0.421, *p* = 0.018, *r*^2^ = 0.177; Figure 3, left) but not in the working context (*r* = −0.112, *p* = 0.541, *r*^2^ = 0.013; Fisher's *z* = 2.12, *p* = 0.034, *q* = 0.561). The proportion of M-A compliments was also negatively correlated with the right-hand 2D4D ratio in the dating context (*r* = −0.392, *p* = 0.029, *r*^2^ = 0.154; Figure 3, right) but not the working context (*r* = −0.071, *p* = 0.699, *r*^2^ = 0.005; Fisher's *z* = −1.29, *p* = 0.197, *q* = 0.343). A General linear regression model showed that art creativity (Beta = 1.433, *t* = 2.592, *p* = 0.015) and right-hand 2D4D ratio (Beta = −273.49, *t* = −2.402, *p* = 0.023) explained 26.7% variance in M-A production, adjusted *R*^2^ = 0.269, *F* = 6.519, *p* = 0.005, *f* ^2^ = 0.368. In the working context, the proportion of L-NA compliments was associated with the scores for K-DOCS science (*r* = 0.358, *p* = 0.044, *r*^2^ = 0.128) but not in the dating context (*r* = −0.134, *p* = 0.473, *r*^2^ = 0.018; Fisher's *z* = −1.92, *p* = 0.055, *q* = 0.509). The proportion of L-A compliments produced was negatively correlated with 2D4D ratios (*r* = −0.562, *p* = 0.001, *r*^2^ = 0.316), but this association was not found in the dating context (*r* = 0.131, *p* = 0.483, *r*^2^ = 0.017; Fisher's *z* = 2.531, *p* = 0.011, *q* = 0.768).

A Process analysis (Hayes, 2013) revealed that context (from dating to working) marginally weakened the association between the K-DOCS art rating and the proportion of M-A compliments ($r_{\text{change}}^{2}=\;0.048$, *p* = 0.059, $r_{\text{pb}}^{2}=$ 0.001) whereas it significantly strengthened that between the K-DOCS science rating and proportion of L-NA compliments (*R*^2^_change_ = 0.063, *p* = 0.041, $r_{\text{pb}}^{2}\;=$ 0.013). In addition, the factor of context was found to negatively moderate the original negative correlation between right-hand 2D4D ratio and L-A production, thus increasing the link strength from dating to working context, *R*^2^_change_ = 0.157, *p* = 0.001, $r_{\text{pb}}^{2}\;=\;0.001$ (see Supplementary Figure S1). No modulatory effect of context was found on the 2D4D ratio correlation with M-A production, *R*^2^_change_ = 0.014, *p* = 0.30, $r_{\text{pb}}^{2}=\;0.001$.

### Women's ratings of male intelligence and relationship potential

The mean intelligence rating for each of the men in the two contexts was computed by averaging the ratings given by the different female judges for each of the individual metaphorical compliments the men produced. Overall the female judges did not rate the perceived intelligence of the male writers differently in the two contexts (mean ± SE rating: dating context = 4.45 ± 0.05, working context = 4.34 ± 0.03, *p* = 0.100, *d* = 0.426). However, only in the dating context was men's perceived intelligence rating by women significantly correlated with their WAIS vocabulary score of verbal IQ (dating context: *r* = 0.410, *p* = 0.030, *r*^2^ = 0.168; working context: *r* = −0.028, *p* = 0.881, *r*^2^ < 0.001), although not their general IQ (Ravens: *p*s > 0.212). The differences between the two contexts did not however quite achieve significance (Fisher's *z* = 1.70, *p* = 0.085, *q* = 0.464). Likewise, only in the dating context was men's perceived intelligence rating by women significantly correlated with the production of M-NA (*r* = 0.38, *p* = 0.035, *r*^2^ = 0.144) and L-A (*r* = −0.436, *p* = 0.014, *r*^2^ = 0.19), but not in working context (with M-NA – *r* = 0.054, *p* = 0.77, *r*^2^ = 0.593; with L-A – *r* = −0.06, *p* = 0.744, *r*^2^ = 0.004). The differences between the two contexts did not achieve significance however (with M-NA: Fisher's *z* = 1.31, *p* = 0.19, *q* = 0.346; with L-A: Fisher's *z* = −1.54, *p* = 0.124, *q* = 0.407). A Process analysis (Hayes, 2013) revealed that context (from dating to working) only marginally weakened both the associations of WAIS vocabulary score ($r_{\text{change}}^{2}=$ 0.050, *p* = 0.07, $r_{\text{pb}}^{2}\;=$ 0.046) and production of M-NA ($r_{\text{change}}^{2}=$ 0.044, *p* = 0.095, $r_{\text{pb}}^{2}=$ 0.070) with the perceived intelligence rating by women. Significant correlations between perceived intelligence and compliment figurativeness (dating context: *r* = 0.786, *p* < 0.001, *r*^2^ = 0.618; working context: *r* = 0.592, *p* < 0.001, *r*^2^ = 0.35; Fisher's *z* = 1.41, *p* = 0.159, *q* = 0.38) and imageability (dating context: *r* = 0.521, *p* = 0.003, *r*^2^ = 0.241; working context: *r* = 0.462, *p* = 0.009, *r*^2^ = 0.213; Fisher's *z* = 1.41, *p* = 0.159, *q* = 0.078) were found in both contexts.

The overall percentage of metaphorical compliments where single women (*N* = 68) chose to have a short- or long-term relationship with the author was low (10.8%), and 75% of them targeted appearance. Importantly though, a significantly higher proportion of men produced at least one compliment in the dating context where women chose to have a romantic relationship with the author (22/31 i.e., 71%) compared to the working context (12/32 i.e., 37.5%; χ^2^ = 7.10, *p* = 0.008, ω = 0.249). Furthermore, the mean perceived intelligence rating of all the metaphorical compliments produced by these same men was significantly higher than for men who did not produce any successful compliments in this respect [mean ± SE rating: romantic selection = 4.45 ± 0.04, non-romantic selection = 4.33 ± 0.04, *t*_(61)_ = 1.999, *p* = 0.05, *d* = 0.504]. A further exploratory analysis of the specific metaphorical compliments where the women raters chose to date the authors revealed again that in general they were inferred to have been produced by more intelligent individuals, and that their quality was higher in terms of appropriateness, figurativeness, imageability, arousal, romance and attractiveness (see Table S8).

## Discussion

The current study aimed to provide experimental support for the “Scheherazade hypothesis” that the evolution of complex language may have been driven to some extent by sexual selection (Miller, 2000). To this end we investigated the differences in language used by men when paying compliments to women in mating (dating) as opposed to non-romantic (working) contexts in order to establish which specific aspects of language form and subject are used in a mating context. Our results revealed that in a mating context, men use complex metaphorical language targeting a woman's appearance more frequently when paying compliments. Furthermore, the frequency of metaphorical compliments produced by men targeting appearance in a mating context was positively associated with their K-DOCS score but negatively associated with their 2D4D ratio. This shows that men producing a higher frequency of this type of compliment were both more creative and had high pT, indicative of greater masculinity. Importantly, women were significantly more likely to want a romantic relationship, and less likely just friendship, with the authors of the metaphorical compliments produced in the mating context. Furthermore, women perceived these later authors as being more intelligent. Thus, overall our findings do provide some support for the “Scheherazade hypothesis” by demonstrating that the language produced by men in a dating context can effectively signal their mate qualities to women.

Our results confirmed our first and main hypothesis that men would produce more and better forms of metaphorical compliments praising women's appearance in a dating rather than a working context. Specifically, men preferred to use M-A compliments in a dating context but L-NA ones in a working one. This suggests that context does indeed modulate male metaphorical language production and/or compliment topic choice and the preference contrast of M-A vs. L-NA also confirms more generally that the type of language used in social communication is context-dependent (Verschueren, 2000). The independent romantic rating of the encounter stories initially provided by the male subjects was positively associated with the production of more M-A and less L-NA compliments. This would suggest that the stronger the initial romantic motivation expressed by the male subjects the more they would attempt to convey this through the type and quality of their compliments. According to the principle of efficiency in language transmission (Grin, 1996), the combination of metaphorical form targeting appearance, may therefore be the most efficient to communicate mate quality and sexual interest. On the other hand a combination of literal compliments which do not target appearance may serve to communicate social support but without conveying sexual interest. Therefore, social praise of women by men using M-A and L-NA type compliments may signal the presence or absence of sexual interest respectively.

Our second hypothesis that use of M-A in a dating context might signal male's mate qualities of creativity and masculinity was also supported by the observation that M-A production frequency was positively correlated with the K-DOCS artistic creativity dimension and negatively with the 2D4D ratio. The GLM regression model further confirmed that men's art creativity and 2D4D ratio were robust predictors for M-A production: the higher art creativity but the lower 2D4D ratio a man had, the more M-A compliments were produced. This is in good agreement with previous studies that creative self-belief could be one of motivations for metaphor production (Karwowski and Lebuda, 2016; McKay et al., 2017) and such self-confidence could also contribute to increased M-A frequency in dating contexts.

Although there is substantial evidence suggesting that metaphorical language is indicative of general creativity (Silvia and Beaty, 2012; Benedek et al., 2014), we only found an association between metaphor production in the dating context and artistic creativity. Similarly, in terms of metaphor quality only figurativeness rather than other language features was associated with compliments produced in the dating context. Art creativity reflects how creative an individual perceives themselves to be in art-related activities, such as visual and architecture design (McKay et al., 2017). Just as with other aesthetic forms of expression such as the visual arts, metaphor is regarded as a literary form of art and demands mental imagery and recruitment of cognitive systems more than sensory-motor systems during their appreciation (Chatterjee and Vartanian, 2014; McQuire et al., 2017). Figurativeness denotes how far language expression deviates from literalness (Thibodeau et al., 2016), and may primarily recruit the right hemisphere of brain in the same way as art, music and emotion (Schmidt and Seger, 2009; Bohrn et al., 2012; Citron and Goldberg, 2014; Jacobs, 2015; Thibodeau et al., 2016). Thus, metaphorical language used in a mating context may particularly signal a man's artistic and emotional faculties, which should also contribute to his overall romantic quality. Interestingly, a recent study has reported that greater creativity can compensate to some extent for low physical attractiveness in a romantic context (Watkins, 2017). Thus, the use of creative language might also serve to enhance the attractiveness of less visually attractive individuals and thereby enhance their mating opportunities.

Additionally, the negative association between a man's 2D4D ratio and the frequency of M-A produced suggests that pT concentrations may influence the use of metaphorical language to signal mate quality. A low 2D4D ratio is indicative of higher pT (Lutchmaya et al., 2004) as well as higher sexual-interest and male specific traits (Klimek et al., 2014; Mailhos et al., 2016). A recent large-scaled study including 89,612 men has also shown significant association between 2D4D ratio and male-type occupations (Manning et al., 2017). Thus, use of metaphorical language to signal mate quality may be exhibited more by men with greater masculine qualities and higher sexual drive as a result of higher pT exposure. While high pT levels have been associated with lower vocabulary size in boys (Kung et al., 2016), no such association has been reported in adults and indeed in our present study we found no correlations between men's general language ability and their 2D4D ratio. The 2D4D ratio has been shown to correlate significantly, but only weakly, with basal testosterone concentrations in men in adulthood (Muller et al., 2011). On the other hand, a stronger relationship may occur in terms of the magnitude of increased testosterone during situations evoking its release, such as aggression or sexual encounters, and this enhanced release is generally indicative of greater masculinity and sex drive (Manning et al., 2014). However, we did not evaluate whether either basal or evoked testosterone levels were higher in men with low 2D4D ratio's in the current study.

Our final hypothesis that if women could interpret mate-quality signals from the compliments produced by men in the dating context then they would be more likely to choose to have a relationship with them and be able to infer their intelligence was also supported. Thus, only from metaphorical compliments produced in the dating context could men's actual verbal IQ, although not general IQ, be communicated effectively to the women raters in terms of perceived intelligence. Furthermore, the metaphorical compliments produced by men targeting appearance in the dating context were significantly more likely to result in women choosing to date the author. This small proportion of the overall number of compliments produced (10.8%) was also rated as inferring greater intelligence and as more arousing, romantic and attractive as well as more appropriate, figurative and imageable. Overall therefore, our findings suggest that men were indeed able to successfully communicate their mate quality to women via the compliments they produced in a dating but not a working context.

Unexpectedly the frequency of L-NA compliments produced in the working context was positively associated with KDOCS science scores. The display of scientific creativity in a working context might well serve to advertise a man's utility as a co-worker to a woman and the deliberate targeting of non-appearance would further emphasize professional and non-sexual aspects. On the other hand, the negative association between the frequency of L-A compliments and the 2D4D ratio in the working context might indicate that men were also potentially seeking to advertise their masculinity indirectly by targeting women's appearance. Masculinity is associated with dominance, which is potentially important in a competitive working environment, and might possibly indicate a strategy whereby men try to advertise their dominance to a female co-worker. However, importantly since they are using literal expression compliments in the working context this may serve to weaken the level of sexual intent conveyed while still displaying dominance. Thus, potentially men may use the language and target of their compliments to women to display appropriate qualities in both mating and working contexts.

There are several limitations in the current study that may have influenced the results obtained. Firstly, the between-subject design used and might have obscured more subtle findings due to the impact of individual heterogeneity, although a within subject design might also have met with different problems with subjects being more conscious in their attempts to distinguish between different contexts. We also ensured that the two groups of male participants were randomly allocated to the two contexts, and matched on range of potential factors that could potentially confound the results. Secondly the use of the 2D4D ratio to infer prenatal or current testosterone levels is a very indirect and potentially unreliable measure and direct measurements would obviously have been preferable (Manning et al., 2014). However, associations between the 2D4D ratio and masculinity and even male attractiveness have been shown (Roney and Maestripieri, 2004). Thirdly, in line with the flirtation literature (Senko and Fyffe, 2010), our findings show that metaphorical compliments targeting appearance in a dating context primarily influence a woman's choice of a short-term rather than a long-term relationship (Penton-Voak et al., 1999; Griskevicius et al., 2006). Thus, other linguistic factors may be of more importance for generating interest in having a long-term relationship. The subjects included in our study were all young students with only a moderate interest in finding a girlfriend and it would therefore be important to replicate our findings in older individuals with a greater motivation to find a partner. Fourthly, in order to focus specifically on communication of mate-quality using language we also used an indirect context that excluded non-verbal aspects and interactions. However, it would be important to investigate whether our findings could extend to a more realistic context of a face-to-face interaction. A further limitation is that we only compared a dating with a working context in the current study and differences observed could be influenced to some extent by different social norms or valued characteristics in the two contexts. It would be important to include other contexts in future, although in the current study the pattern of specific features that men targeted with their compliments on either appearance or non-appearance were very similar in the two contexts (e.g., no highly sex-loaded compliments targeting breasts or buttocks were produced in the dating context which would be considered inappropriate in a working context). A final limitation is that the subject numbers used in the study resulted in observed statistical power of <80% for some of the analyses, although in general effect sizes were medium to large in size. Larger future studies may therefore be needed to replicate some of our findings.

In conclusion, this study has provided experimental support for the theory that a figurative form of language use in a mating context may have evolved to signal of creativity, intelligence and masculinity displayed by men and can be detected by women during social interactions. However, both language form and topic clearly play a complex and interactive role in signaling both male qualities and intentions. More studies in the future are required to explore the implicit information transmission of mate quality through the form and topic of language in real contexts.

## Author contributions

All authors approved the final manuscript and contributed to the experiment. The first author designed the research and compiled program and drafted the manuscript; QY carried out the experiments and participated in data analysis; XM jointly designed the experiment and helped with manuscript writing. BB helped with data interpretation and proofreading. KL and FZ helped with part of data analysis. KK revised the manuscript.

### Conflict of interest statement

The authors declare that the research was conducted in the absence of any commercial or financial relationships that could be construed as a potential conflict of interest. The reviewer, FK, and handling editor declared their shared affiliation.
