# Supplementary material for: Men Who Compliment a Woman's Appearance Using Metaphorical Language: Associations with Creativity, Masculinity, Intelligence and Attractiveness
Source: Front Psychol. 2017 Dec 21;8:2185. doi: 10.3389/fpsyg.2017.02185 (PMC5742614; doi:10.3389/fpsyg.2017.02185)
Supplement: Supplementary file 1 [file Table1.DOCX]

Supplementary Material

Men who compliment a woman’s appearance using metaphorical language: associations with creativity, 2D4D ratio and attractiveness

**Zhao Gao, Qi Yang, Xiaole Ma, Benjamin Becker, Keshuang Li, Feng Zhou, Keith M. Kendrick ***

*** Correspondence:** Keith M. Kendrick: [k.kendrick.uestc@gmail.com](mailto:k.kendrick.uestc@gmail.com)

| **Table S1**  Relationship status (RS) homogeneity test between contexts. (N=63) | | |
| --- | --- | --- |
| *Phi*=0.165 | Contexts | |
|  | Dating (N=31) | Working  (N=32) |
| \| Having a girlfriend \| Count \| 16 \| 22 \| \| --- \| --- \| --- \| --- \| | | |
| \| \|  \| % within Context \| 51.6% \| 68.8% \| \| --- \| --- \| --- \| --- \| \|  \| % within RS \| 42.1% \| 60.0% \| \| \| --- \| --- \| --- \| --- \| --- \| --- \| --- \| --- \| --- \| | | |
| \| Having no girlfriend \| Count \| 15 \| 10 \| \| --- \| --- \| --- \| --- \| | | |
| \| \|  \| % within Context \| 48.4% \| 31.3% \| \| --- \| --- \| --- \| --- \| \|  \| % within RS \| 57.9% \| 40.0% \| \| \| --- \| --- \| --- \| --- \| --- \| --- \| --- \| --- \| --- \| | | |
|  | | |
