# Supplementary material for: Men Who Compliment a Woman's Appearance Using Metaphorical Language: Associations with Creativity, Masculinity, Intelligence and Attractiveness
Source: Front Psychol. 2017 Dec 21;8:2185. doi: 10.3389/fpsyg.2017.02185 (PMC5742614; doi:10.3389/fpsyg.2017.02185)
Supplement: Supplementary file 2 [file Table2.docx]

***Supplementary Material***

**Men who compliment a woman’s appearance using metaphorical language: associations with creativity, 2D4D ratio and attractiveness**

**Zhao Gao, Qi Yang, Xiaole Ma, Benjamin Becker, Keshuang Li, Feng Zhou, Keith M. Kendrick ***

*** Correspondence:** Keith M. Kendrick: [k.kendrick.uestc@gmail.com](mailto:k.kendrick.uestc@gmail.com)

| **Table S2**  Desirability to start a relationship of male participants in two contexts. | | | | | | | | | |
| --- | --- | --- | --- | --- | --- | --- | --- | --- | --- |
|  | Dating Context | | |  | Working Context | | | *t* | *p* |
|  | N | Mean | SE |  | N | Mean | SE |  |  |
| Having a girlfriend | 16 | 3.63 | 0.22 |  | 22 | 3.73 | 0.18 | -0.37 | 0.72 |
|  |  |  |  |  |  |  |  |  |  |
| Having no girlfriend | 15 | 3.80 | 0.28 |  | 10 | 3.80 | 0.41 | 0.00 | 1.00 |
|  | | | | | | | | | |
