# Supplementary material for: Men Who Compliment a Woman's Appearance Using Metaphorical Language: Associations with Creativity, Masculinity, Intelligence and Attractiveness
Source: Front Psychol. 2017 Dec 21;8:2185. doi: 10.3389/fpsyg.2017.02185 (PMC5742614; doi:10.3389/fpsyg.2017.02185)
Supplement: Supplementary file 3 [file Table3.DOCX]

Supplementary Material

Men who compliment a woman’s appearance using metaphorical language: associations with creativity, 2D4D ratio and attractiveness

**Zhao Gao, Qi Yang, Xiaole Ma, Benjamin Becker, Keshuang Li, Feng Zhou, Keith M. Kendrick ***

*** Correspondence:** Keith M. Kendrick: [k.kendrick.uestc@gmail.com](mailto:k.kendrick.uestc@gmail.com)

| **Table S3**  The homogeneity test of writing context and love attitude scale (LAS) types between two contexts. | | | |
| --- | --- | --- | --- |
|  | | Contexts | |
|  |  | Dating (N=31) | Working  (N=32) |
| Feel type (Cramer’s *V*=0.300) | | | |
| *Storge* | Count | 10 | 12 |
|  | % within Context | 32.3% | 37.5% |
|  | % within LAS_feel | 45.5% | 57.1% |
| *Eros* | Count | 20 | 16 |
|  | % within Context | 64.5% | 50.0% |
|  | % within LAS_feel | 55.6% | 45.7% |
| *Ludus* | Count | 1 | 4 |
|  | % within Context | 3.2% | 12.5% |
|  | % within LAS_feel | 20.0% | 80.0% |
|  |  |  |  |
| Act type (Cramer’s *V*=0.515) | | | |
| *Pragma* | Count | 5 | 9 |
|  | % within Context | 16.1% | 28.1% |
|  | % within LAS_act | 35.7% | 64.3% |
| *Mania* | Count | 2 | 2 |
|  | % within Context | 6.5% | 6.3% |
|  | % within LAS_act | 50.0% | 50.0% |
| *Agape* | Count | 24 | 21 |
|  | % within Context | 77.4% | 65.6% |
|  | % within LAS_act | 53.3% | 48.8% |
|  | | | |
