# Supplementary material for: Men Who Compliment a Woman's Appearance Using Metaphorical Language: Associations with Creativity, Masculinity, Intelligence and Attractiveness
Source: Front Psychol. 2017 Dec 21;8:2185. doi: 10.3389/fpsyg.2017.02185 (PMC5742614; doi:10.3389/fpsyg.2017.02185)
Supplement: Supplementary file 4 [file Table4.docx]

Supplementary Material

Men who compliment a woman’s appearance using metaphorical language: associations with creativity, 2D4D ratio and attractiveness

**Zhao Gao, Qi Yang, Xiaole Ma, Benjamin Becker, Keshuang Li, Feng Zhou, Keith M. Kendrick ***

*** Correspondence:** Keith M. Kendrick: [k.kendrick.uestc@gmail.com](mailto:k.kendrick.uestc@gmail.com)

**Table S4**

The distribution of choices of the 30 different stimulus pictures and the average ratings for them.

| Picture No. | Contexts | Frequency | | Chi Square | Average Rating^a^ | | | | |
| --- | --- | --- | --- | --- | --- | --- | --- | --- | --- |
|  |  | Selected | Not selected |  | AG | AR | AT | IN | VA |
| 1 | Dating | 6 | 24 | .472 | 5.67 | 6.44 | 6.56 | 4.78 | 6.22 |
|  | Working | 3 | 27 |  |  |  |  |  |  |
| 2 | Dating | 5 | 25 | 0.748 | 3.92 | 5.25 | 5.25 | 5.67 | 4.58 |
|  | Working | 7 | 23 |  |  |  |  |  |  |
| 3 | Dating | 4 | 26 | 0.112 | 5.00 | 5.75 | 5.75 | 5.25 | 5.50 |
|  | Working | 0 | 30 |  |  |  |  |  |  |
| 4 | Dating | 2 | 28 | 1.000 | 5.00 | 4.25 | 4.50 | 5.25 | 5.25 |
|  | Working | 2 | 28 |  |  |  |  |  |  |
| 5 | Dating | 1 | 29 | 0.103 | 5.71 | 6.00 | 5.29 | 5.29 | 6.57 |
|  | Working | 6 | 24 |  |  |  |  |  |  |
| 6 | Dating | 7 | 23 | 0.567 | 5.53 | 5.18 | 5.47 | 5.47 | 6.47 |
|  | Working | 10 | 20 |  |  |  |  |  |  |
| 7 | Dating | 2 | 28 | 0.492 | 5.50 | 6.00 | 5.50 | 5.00 | 5.00 |
|  | Working | 0 | 30 |  |  |  |  |  |  |
| 8 | Dating | 0 | 30 | 0.112 | 5.00 | 5.75 | 5.75 | 5.25 | 5.00 |
|  | Working | 4 | 26 |  |  |  |  |  |  |
| 9 | Dating | 2 | 28 | 0.671 | 5.17 | 5.67 | 5.83 | 5.33 | 4.83 |
|  | Working | 4 | 26 |  |  |  |  |  |  |
| 10 | Dating | 11 | 19 | 0.252 | 5.00 | 6.00 | 5.71 | 5.12 | 5.24 |
|  | Working | 6 | 24 |  |  |  |  |  |  |
| 11 | Dating | 4 | 26 | 0.072 | 4.33 | 4.67 | 4.67 | 5.47 | 4.47 |
|  | Working | 11 | 19 |  |  |  |  |  |  |
| 12 | Dating | 6 | 24 | 1.000 | 4.82 | 5.82 | 5.82 | 5.55 | 5.09 |
|  | Working | 5 | 25 |  |  |  |  |  |  |
| 13 | Dating | 4 | 26 | 0.112 | 3.75 | 4.00 | 5.00 | 4.75 | 2.75 |
|  | Working | 0 | 30 |  |  |  |  |  |  |
| 14 | Dating | 1 | 29 | 0.103 | 5.00 | 4.71 | 4.57 | 5.29 | 4.86 |
|  | Working | 6 | 24 |  |  |  |  |  |  |
| 15 | Dating | 0 | 30 | 1.000 | 4.00 | 6.00 | 6.00 | 4.00 | 4.00 |
|  | Working | 1 | 29 |  |  |  |  |  |  |
| 16 | Dating | 0 | 30 | 1.000 | 3.00 | 4.00 | 4.00 | 7.00 | 4.00 |
|  | Working | 1 | 29 |  |  |  |  |  |  |
| 17 | Dating | 3 | 27 | **0.015*** | 4.47 | 5.33 | 5.40 | **5.53** | 4.73 |
|  | Working | 12 | 18 |  |  |  |  |  |  |
| 18 | Dating | 1 | 29 | 0.195 | 5.00 | 4.50 | 4.50 | 4.17 | 5.17 |
|  | Working | 5 | 25 |  |  |  |  |  |  |
| 19 | Dating | 2 | 28 | 1.000 | 3.50 | 6.25 | 6.00 | 5.00 | 3.75 |
|  | Working | 2 | 28 |  |  |  |  |  |  |
| 20 | Dating | 10 | 20 | **0.021*** | 5.25 | 5.83 | **5.67** | 4.92 | 4.83 |
|  | Working | 2 | 28 |  |  |  |  |  |  |
| 21 | Dating | 2 | 28 | 1.000 | 5.33 | 5.67 | 5.67 | 5.67 | 6.00 |
|  | Working | 1 | 29 |  |  |  |  |  |  |
| 22 | Dating | 8 | 22 | 1.000 | 5.18 | 5.94 | 5.82 | 5.82 | 5.47 |
|  | Working | 9 | 21 |  |  |  |  |  |  |
| 23 | Dating | 1 | 29 | 1.000 | 4.33 | 5.33 | 5.33 | 4.67 | 4.00 |
|  | Working | 2 | 28 |  |  |  |  |  |  |
| 24 | Dating | 16 | 14 | **0.001**** | 4.32 | 6.11 | **6.26** | 5.05 | 4.16 |
|  | Working | 3 | 27 |  |  |  |  |  |  |
| 25 | Dating | 1 | 29 | 1.000 | 1.50 | 4.00 | 5.00 | 6.00 | 3.50 |
|  | Working | 1 | 29 |  |  |  |  |  |  |
| 26 | Dating | 0 | 30 | 0.112 | 4.75 | 5.50 | 5.00 | 4.25 | 5.25 |
|  | Working | 4 | 26 |  |  |  |  |  |  |
| 27 | Dating | 5 | 25 | 1.000 | 5.91 | 5.91 | 6.00 | 4.73 | 6.00 |
|  | Working | 6 | 24 |  |  |  |  |  |  |
| 28 | Dating | 2 | 28 | 1.000 | 4.33 | 5.00 | 4.33 | 3.33 | 5.00 |
|  | Working | 1 | 29 |  |  |  |  |  |  |
| 29 | Dating | 7 | 23 | 0.748 | 5.58 | 5.75 | 5.92 | 5.67 | 5.67 |
|  | Working | 5 | 25 |  |  |  |  |  |  |
| 30 | Dating | 7 | 23 | **0.052*** | 4.75 | 5.75 | **5.75** | 4.13 | 4.00 |
|  | Working | 1 | 29 |  |  |  |  |  |  |
| 1. AG=agreeableness, AR=arousal, AT=attractiveness, IN=intelligence, VA=valence   * Significant at *p=*0.052 two tailed, ** *p*<0.05 | | | | | | | | | |
