# Supplementary material for: Men Who Compliment a Woman's Appearance Using Metaphorical Language: Associations with Creativity, Masculinity, Intelligence and Attractiveness
Source: Front Psychol. 2017 Dec 21;8:2185. doi: 10.3389/fpsyg.2017.02185 (PMC5742614; doi:10.3389/fpsyg.2017.02185)
Supplement: Supplementary file 5 [file Table5.docx]

Supplementary Material

Men who compliment a woman’s appearance using metaphorical language: associations with creativity, 2D4D ratio and attractiveness

**Zhao Gao, Qi Yang, Xiaole Ma, Benjamin Becker, Keshuang Li, Feng Zhou, Keith M. Kendrick ***

*** Correspondence:** Keith M. Kendrick: [k.kendrick.uestc@gmail.com](mailto:k.kendrick.uestc@gmail.com)

**Table S5**

The three photos of women that were selected differentially in the dating and working contexts due to their higher attractiveness (see Table S5) did not contribute significantly to the overall difference in metaphorical compliment production found in the two contexts. Analysis shows that results remain significant even when men choosing pictures 20/24/30 were excluded.

| Number | Dating  (N=18) | |  | Working  (N=30) | |
| --- | --- | --- | --- | --- | --- |
|  |  |  |  |  |  |
|  | Mean | SE |  | Mean | SE |
| M-A | 12.50 | 1.34 |  | 6.62 | 1.05 |
| M-NA | 2.89 | 0.90 |  | 5.18 | 0.69 |
| L-A | 3.14 | 0.56 |  | 2.67 | 0.43 |
| L-NA | 3.11 | 1.15 |  | 5.73 | 0.89 |
|  |  |  |  |  |  |
| 2x4 ANOVA | | | | | |
|  |  | F | df | Sig. | Partial eta square |
| Compliment type |  | 20.23 | 3 | **< 0.001** | 0.31 |
| Context x Compliment type |  | 8.98 | 3 | **< 0.001** | 0.16 |
|  |  |  |  |  |  |
