# Supplementary material for: Men Who Compliment a Woman's Appearance Using Metaphorical Language: Associations with Creativity, Masculinity, Intelligence and Attractiveness
Source: Front Psychol. 2017 Dec 21;8:2185. doi: 10.3389/fpsyg.2017.02185 (PMC5742614; doi:10.3389/fpsyg.2017.02185)
Supplement: Supplementary file 6 [file Table6.docx]

Supplementary Material

Men who compliment a woman’s appearance using metaphorical language: associations with creativity, 2D4D ratio and attractiveness

**Zhao Gao, Qi Yang, Xiaole Ma, Benjamin Becker, Keshuang Li, Feng Zhou, Keith M. Kendrick ***

*** Correspondence:** Keith M. Kendrick: [k.kendrick.uestc@gmail.com](mailto:k.kendrick.uestc@gmail.com)

**Table S6**

Break down of specific targets of compliments included in the general categories of: “Nonappearance” and “Appearance”. The proportion of topic subcategories distributed within each context and Chi-square test result for the difference between contexts.

| Topic subcategory | Proportion within dating context (%) | Proportion within working context (%) | Chi-square | *p* value |
| --- | --- | --- | --- | --- |
| *Nonappearance* | | | | |
| Mind | 36.36 | 29.91 | 7.571 | 0.109 |
| Personality | 34.85 | 49.57 |  |  |
| Ability | 1.52 | 5.13 |  |  |
| Temperament | 10.61 | 7.69 |  |  |
| General | 16.67 | 7.69 |  |  |
|  | | | | |
| *Appearance* | | | | |
| Smile | 12.84 | 19.50 | 15.556 | 0.744 |
| Face | 8.72 | 8.81 |  |  |
| Hand | 4.13 | 2.52 |  |  |
| Eye | 16.97 | 17.61 |  |  |
| Hair | 9.63 | 8.81 |  |  |
| Body | 6.42 | 5.03 |  |  |
| Voice | 6.88 | 9.43 |  |  |
| Skin | 2.75 | 2.52 |  |  |
| Action & expression | 8.72 | 4.40 |  |  |
| Gait | 0.00 | 0.63 |  |  |
| Lip | 5.96 | 6.29 |  |  |
| Nose | 0.92 | 0.63 |  |  |
| Upper body | 1.83 | 1.89 |  |  |
| Eyebrow | 5.96 | 3.77 |  |  |
| Teeth | 0.92 | 1.89 |  |  |
| Foot | 0.46 | 0.00 |  |  |
| Chin | 0.46 | 0.00 |  |  |
| Scent | 0.46 | 0.00 |  |  |
| Clothes & accessories | 1.38 | 3.14 |  |  |
| Overall appearance | 2.75 | 1.89 |  |  |
| Ears | 1.83 | 1.26 |  |  |
