# Supplementary material for: Men Who Compliment a Woman's Appearance Using Metaphorical Language: Associations with Creativity, Masculinity, Intelligence and Attractiveness
Source: Front Psychol. 2017 Dec 21;8:2185. doi: 10.3389/fpsyg.2017.02185 (PMC5742614; doi:10.3389/fpsyg.2017.02185)
Supplement: Supplementary file 7 [file Table7.docx]

Supplementary Material

Men who compliment a woman’s appearance using metaphorical language: associations with creativity, 2D4D ratio and attractiveness

**Zhao Gao, Qi Yang, Xiaole Ma, Benjamin Becker, Keshuang Li, Feng Zhou, Keith M. Kendrick ***

*** Correspondence:** Keith M. Kendrick: [k.kendrick.uestc@gmail.com](mailto:k.kendrick.uestc@gmail.com)

**Table S7**

The ANOVA results of scores of 18 compliments rated by all of the women across the four rating groups. No statistical differences were found between women in the four groups.

| Criteria | Group | Rater Number | Mean | SE | df | *F* | *p* |
| --- | --- | --- | --- | --- | --- | --- | --- |
| Appropriateness | 1 | 30 | 4.66 | 0.10 | 3 | 0.43 | 0.73 |
|  | 2 | 30 | 4.50 | 0.18 |  |  |  |
|  | 3 | 30 | 4.53 | 0.17 |  |  |  |
|  | 4 | 24 | 4.48 | 0.16 |  |  |  |
| Valence | 1 | 30 | 5.27 | 0.11 | 3 | 0.30 | 0.83 |
|  | 2 | 30 | 5.35 | 0.15 |  |  |  |
|  | 3 | 30 | 5.22 | 0.17 |  |  |  |
|  | 4 | 24 | 5.20 | 0.17 |  |  |  |
| Figurativeness | 1 | 30 | 4.86 | 0.17 | 3 | 1.16 | 0.33 |
|  | 2 | 30 | 4.58 | 0.22 |  |  |  |
|  | 3 | 30 | 4.76 | 0.20 |  |  |  |
|  | 4 | 24 | 4.47 | 0.21 |  |  |  |
| Familiarity | 1 | 30 | 4.37 | 0.14 | 3 | 0.93 | 0.43 |
|  | 2 | 30 | 4.28 | 0.16 |  |  |  |
|  | 3 | 30 | 4.23 | 0.15 |  |  |  |
|  | 4 | 24 | 4.52 | 0.21 |  |  |  |
| Imageability | 1 | 30 | 3.91 | 0.13 | 3 | 1.91 | 0.13 |
|  | 2 | 30 | 3.63 | 0.20 |  |  |  |
|  | 3 | 30 | 3.98 | 0.16 |  |  |  |
|  | 4 | 24 | 4.09 | 0.21 |  |  |  |
| Arousal | 1 | 30 | 4.61 | 0.16 | 3 | 1.04 | 0.38 |
|  | 2 | 30 | 4.44 | 0.17 |  |  |  |
|  | 3 | 30 | 4.40 | 0.18 |  |  |  |
|  | 4 | 24 | 4.27 | 0.15 |  |  |  |
| Romance | 1 | 30 | 4.40 | 0.22 | 3 | 0.53 | 0.67 |
|  | 2 | 30 | 4.19 | 0.23 |  |  |  |
|  | 3 | 30 | 4.16 | 0.23 |  |  |  |
|  | 4 | 24 | 4.11 | 0.19 |  |  |  |
| Attractiveness | 1 | 30 | 4.45 | 0.18 | 3 | 0.79 | 0.50 |
|  | 2 | 30 | 4.31 | 0.20 |  |  |  |
|  | 3 | 30 | 4.19 | 0.22 |  |  |  |
|  | 4 | 24 | 4.14 | 0.16 |  |  |  |
| Intelligence | 1 | 30 | 4.53 | 0.12 | 3 | 1.01 | 0.39 |
|  | 2 | 30 | 4.44 | 0.17 |  |  |  |
|  | 3 | 30 | 4.62 | 0.21 |  |  |  |
|  | 4 | 24 | 4.30 | 0.13 |  |  |  |
