# Supplementary material for: Men Who Compliment a Woman's Appearance Using Metaphorical Language: Associations with Creativity, Masculinity, Intelligence and Attractiveness
Source: Front Psychol. 2017 Dec 21;8:2185. doi: 10.3389/fpsyg.2017.02185 (PMC5742614; doi:10.3389/fpsyg.2017.02185)
Supplement: Supplementary file 8 [file Table8.docx]

Supplementary Material

Men who compliment a woman’s appearance using metaphorical language: associations with creativity, 2D4D ratio and attractiveness

**Zhao Gao, Qi Yang, Xiaole Ma, Benjamin Becker, Keshuang Li, Feng Zhou, Keith M. Kendrick ***

*** Correspondence:** Keith M. Kendrick: [k.kendrick.uestc@gmail.com](mailto:k.kendrick.uestc@gmail.com)

**Table S8**

Ratings of the language used in compliments selected by 68 single women to establish a romantic relation with the author (34 different men). These compliments were rated across all the criteria used, other than familiarity. Notably inferred intelligence of the author was also significantly higher.

| Criteria | Selected male group (N=60) | |  | Not selected male group (N=490) | | *t* | *p* |
| --- | --- | --- | --- | --- | --- | --- | --- |
|  | Mean | SE |  | Mean | SE |  |  |
| Appropriateness | 4.69 | 0.10 |  | 4.39 | 0.03 | 2.914 | **0.004** |
| Valence | 5.26 | 0.08 |  | 5.10 | 0.02 | 2.143 | **0.033** |
| Figurativeness | 4.80 | 0.08 |  | 4.45 | 0.03 | 4.285 | **< 0.001** |
| Familiarity | 4.15 | 0.12 |  | 4.25 | 0.04 | -0.844 | 0.399 |
| Imageability | 4.12 | 0.09 |  | 3.66 | 0.03 | 5.616 | **<0.001** |
| Arousal | 4.86 | 0.08 |  | 4.24 | 0.02 | 7.203 | **< 0.001** |
| Romance | 4.71 | 0.11 |  | 3.92 | 0.03 | 8.089 | **< 0.001** |
| Attractiveness | 4.72 | 0.09 |  | 4.10 | 0.02 | 8.016 | **< 0.001** |
|  |  |  |  |  |  |  |  |
| Intelligence inferred | 4.86 | 0.07 |  | 4.31 | 0.02 | 8.360 | **< 0.001** |
| Note: These results should be interpreted with caution since this is only an exploratory analysis with such a large difference in the sentence numbers in the two groups. | | | | | | | |
