# Supplementary material for: Men Who Compliment a Woman's Appearance Using Metaphorical Language: Associations with Creativity, Masculinity, Intelligence and Attractiveness
Source: Front Psychol. 2017 Dec 21;8:2185. doi: 10.3389/fpsyg.2017.02185 (PMC5742614; doi:10.3389/fpsyg.2017.02185)
Supplement: Supplementary file 9 [file DataSheet1.docx]

Supplementary Material

Men who compliment a woman’s appearance using metaphorical language: associations with creativity, 2D4D ratio and attractiveness

**Zhao Gao, Qi Yang, Xiaole Ma, Benjamin Becker, Keshuang Li, Feng Zhou, Keith M. Kendrick ***

*** Correspondence:** Keith M. Kendrick: [k.kendrick.uestc@gmail.com](mailto:k.kendrick.uestc@gmail.com)

**File S1 Instructions for the scenarios in the dating and working contexts.**

*Thank you for having rated the 30 college female students, whose photos are provided by our cooperator, an on-line dating company. They are between 18-22 years old and ready to start a relationship. Now imagine you are going to date four of these women, which ones would you choose? Please select their photos.* (Dating Scenario)

*Thank you for having rated the 30 college female students, whose photos are provided by our cooperator, an on-line part-time job agency. They are between 18-22 years old and all getting steady with their boyfriends. Now they need to join in a work team for a science project. Imagine you are going to need four women co-workers in your project. Which four of these women would you choose to work with? Please select their photos.* (Working Scenario)
