# Supplementary material for: Men Who Compliment a Woman's Appearance Using Metaphorical Language: Associations with Creativity, Masculinity, Intelligence and Attractiveness
Source: Front Psychol. 2017 Dec 21;8:2185. doi: 10.3389/fpsyg.2017.02185 (PMC5742614; doi:10.3389/fpsyg.2017.02185)
Supplement: Supplementary file 10 [file Image1.PDF]

Supplementary Material

Men who compliment a woman's appearance using metaphorical language: associations with creativity, 2D4D ratio and attractiveness

Zhao Gao, Qi Yang, Xiaole Ma, Benjamin Becker, Keshuang Li, Feng Zhou, Keith M. Kendrick *

*** Correspondence:** Keith M. Kendrick: k.kendrick.uestc@gmail.com

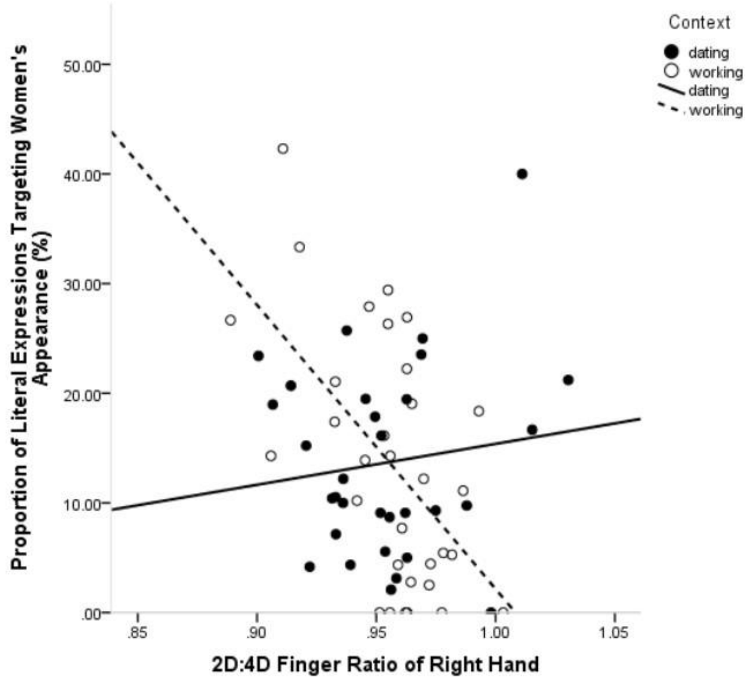

Supplementary Figure S1. Process analysis (Hays, 2013) showed that context negatively mediated the negative association between the production of literal expression targeting women's appearance and the male producers' 2D:4D ratio of right hand. Coefficient=-296.37±86.58, t=-3.423, p=0.001.
